# Supplementary material for: Therapeutic Targeting of Stat3 Using Lipopolyplex Nanoparticle-Formulated siRNA in a Syngeneic Orthotopic Mouse Glioma Model
Source: Cancers (Basel). 2019 Mar 8;11(3):333. doi: 10.3390/cancers11030333 (PMC6468565; doi:10.3390/cancers11030333)
Supplement: Supplementary file 1 [file cancers-11-00333-s001.pdf]

Supplementary Materials: Therapeutic targeting of Stat3 using lipopolyplex nanoparticle-formulated siRNA in a syngeneic orthotopic mouse glioma model

Benedikt Linder, Ulrike Weirauch, Alexander Ewe, Anja Uhmann, Volker Seifert, Michel Mittelbronn, Patrick N. Harter, Achim Aigner and Donat Kögel

Supplementary Methods:

Cell cycle analysis with Nocodazole-induced G2/M-block

Cell cycle distribution in U87 and Mz18 cells was analyzed following the same protocol; however, 100 ng/ml nocodazole was added 24 h after transfection for inducing a G2/M arrest. Thus, smaller fractions of cells reaching the G2/M block at 8 h after nocodazole addition indicate slower cell cycle progression.

Supplementary Figures:

a

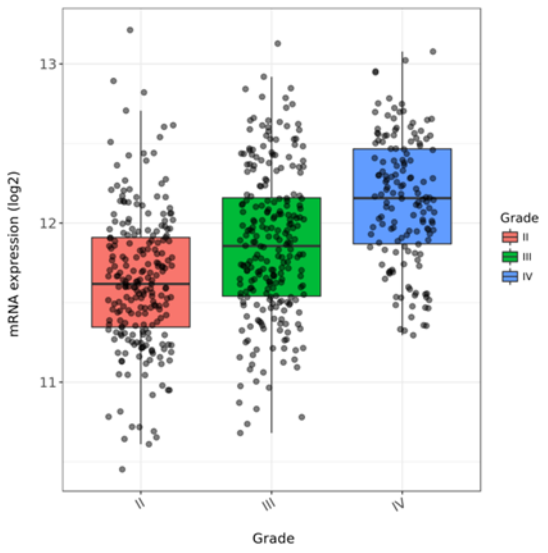

b

Comparison	diff	lwr	upr	p.adj	Significance
IV-II	0.50	0.39	0.61	0.00	***
IV-III	0.27	0.16	0.38	0.00	***
III-II	0.23	0.13	0.32	0.00	***

Figure S1. STAT3-mRNA expression from the TCGA dataset GBMLGG [62] plotted using the GlioVis portal (gliovis.bioinfo.cnio.es [63]). (**a**) Box-Plots depicting the expression levels of STAT3-mRNA in different glioma subtypes. (**b**) Statistical comparison of STAT3-mRNA expression using Tukey HSD-test as provided via the GlioVis-Portal. The table shows the difference between pairs, the 95% confidence interval and the p-value of the pairwise comparisons. *** $p < 0.001$; ns, not significant.

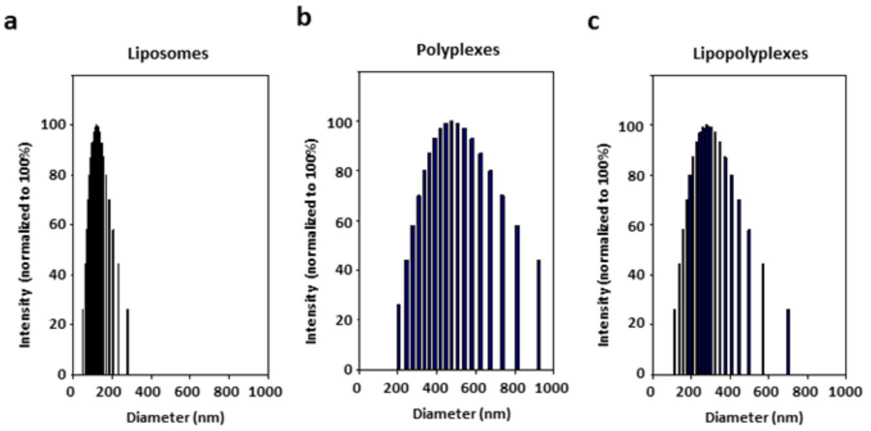

Figure S2. Nanoparticle sizes as determined by Zetasizer (dynamic light scattering) (**a**–**c**) Results of size measurements by Zetasizer. Two independent samples were measured 10 times.

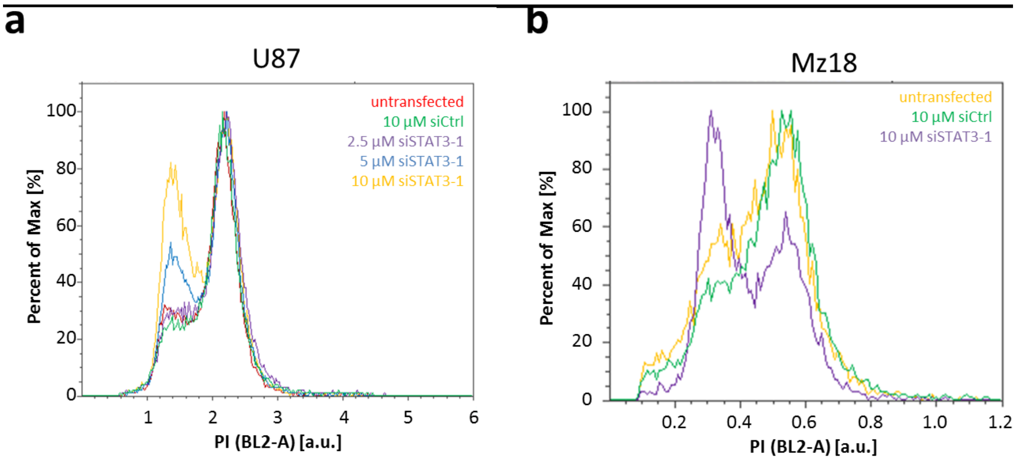

Figure S3. siStat3 hinders cell cycle progression in human GBM cell lines. (**a** and **b**) Cell cycle analysis in (**a**) U87 and (**b**) Mz18 cells after transfection with siRNA as indicated in the figures. Note that nocodazole (100 ng/ml) was added 48 h after transfection for inducing a G2/M arrest. Thus, larger percentages of cells in G0/G1 indicate smaller fractions of cells reaching the G2/M block at 16 h after nocodazole addition and thus slower cell cycle progression. In Figure S3a, an siSTAT3 dose-dependent decrease in cell cycle deceleration can be observed.

© 2019 by the authors. Licensee MDPI, Basel, Switzerland. This article is an open access article distributed under the terms and conditions of the Creative Commons Attribution (CC BY) license (http://creativecommons.org/licenses/by/4.0/)
